# Postnatally Acquired Neonatal CMV Infection in Preterm Infants: From a Case Series to a Narrative Review of the Literature

**DOI:** 10.3390/children13010046

**Published:** 2025-12-29

**Authors:** Serena Salomè, Ida D’Acunzo, Clara Coppola, Giovanna Montesano, Gaetano Ausanio, Angela Umbaldo, Fiorella Migliaro, Letizia Capasso, Francesco Raimondi

**Affiliations:** 1 Division of Neonatology, Department of Translational Medical Sciences, University of Naples Federico II, 80131 Naples, Italy; 2 Division of Neonatology, Azienda Ospedaliera S. Anna e S. Sebastiano, 81100 Caserta, Italy

**Keywords:** postnatal cytomegalovirus infection, CMV, pCMV

## Abstract

**Highlights:**

**What are the main findings?**

- In high-risk preterm infants, postnatally acquired CMV can lead to severe disease, including sepsis-like syndrome, pneumonia, cytopenias, hepatitis, and colitis, with the potential for long-term sequelae or death.
- Clinical management remains highly variable, with substantial differences in diagnostic strategies, therapeutic approaches, and breastmilk handling across centers and countries.

**What are the implications of the main findings?**

- Postnatal CMV infection remains under-recognized, highlighting the need for further research and clearer clinical guidelines to optimize neonatal outcomes.
- Harmonized, evidence-based recommendations on diagnosis, treatment, and breastmilk management are needed to improve care for at-risk or affected preterm infants.

**Abstract:**

**Background:** Postnatal cytomegalovirus (pCMV) infection is a frequent viral condition in early infancy and is primarily acquired through maternal breastfeeding. Although usually asymptomatic in term infants, it can lead to significant morbidity in preterm neonates (gestational age < 32 weeks) and in those with very low birthweight (<1500 g), presenting with sepsis-like syndrome, pneumonia, cytopenia, hepatitis, or colitis. Severe cases may result in long-term sequelae or death. **Objectives:** To describe a series of cases of pCMV infection and review the current evidence on its epidemiology, clinical manifestations, outcomes, and therapeutic management, aiming to identify gaps in knowledge and propose opportunities for improving the care of preterm infants. Methods: We analyzed clinical presentations of pCMV disease in a case series of preterm infants and reported cases and reviewed the recent literature regarding diagnostic approaches, antiviral therapy, and strategies for breastmilk management. **Results:** Current data highlight substantial variability in clinical management and outcomes. The lack of consensus on antiviral indications and treatment duration reflects a limited understanding of the disease’s natural history. Approaches to breastmilk handling differ widely among centers and countries, further complicating the standardization of care. **Conclusions:** pCMV infection remains a relevant yet under-recognized condition in neonatal medicine. Improved diagnostic strategies, clearer therapeutic guidelines, and harmonized recommendations for breastmilk management are needed to optimize the care of preterm infants at risk of or affected by pCMV disease.

## 1. Introduction

Postnatal cytomegalovirus (pCMV) infection represents one of the most frequent viral infections during early infancy, with breastmilk recognized as the primary route of transmission [1]. In term infants, pCMV infection is generally asymptomatic; however, very preterm neonates (gestational age < 32 weeks) and those with very low birthweight (VLBW, <1500 g) are at greater risk of developing symptomatic disease. This vulnerability is primarily related to their limited transplacental transfer of maternal antibodies and immature immune response, which together reduce their ability to control viral replication [2].

Clinically, pCMV infection in preterm infants can manifest as sepsis-like syndrome, pneumonia, thrombocytopenia, neutropenia, hepatitis, or colitis, often complicating the neonatal course and worsening overall prognosis [3]. Beyond acute disease, symptomatic pCMV infection has been associated with long-term pulmonary and neurodevelopmental sequelae, although large-scale prospective studies are still required to accurately define these outcomes [4]. Importantly, distinguishing the specific contribution of pCMV from the comorbidities related to prematurity remains a persistent clinical challenge.

Despite clear evidence that breastmilk is the main source of infection, management of milk handling and treatment remain heterogeneous worldwide. Furthermore, the therapeutic role of antiviral agents in pCMV disease is still debated, with uncertainties surrounding treatment indications and the timing, and duration of treatment.

Overall, significant gaps remain in our understanding of the natural history and optimal management of pCMV infection in preterm infants, both during the acute phase and in long-term follow-up. Starting from a series of clinical cases, this narrative review aims to summarize current evidence on epidemiology, clinical presentation, outcomes, and therapeutic strategies. It also discusses emerging strategies to minimize CMV transmission through breastmilk and to improve diagnostic and management approaches in this highly vulnerable population.

## 2. Epidemiology

**Figure 1:**
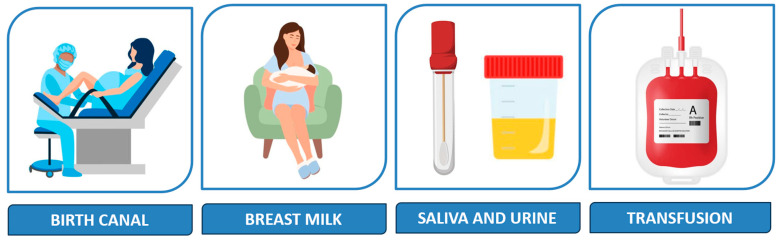
Main postnatal sources of infection.

CMV is the most common cause of perinatally acquired infection worldwide [5], and mother-to-child transmission in the postnatal period occurs primarily but not exclusively via breastmilk [6,7,8], as shown in Figure 1.

Transmission of CMV in the postnatal period may occur intrapartum, via aspiration of infected cervical secretions (33%), or after birth, through contact with infected fluids such as breastmilk (38%), saliva and urine (29%) [1], or blood products [9]. Prolonged rupture of membranes appears to increase the likelihood of early CMV acquisition [7].

CMV transmission via breastmilk (BM) occurs frequently, with incidence estimates influenced by population characteristics and diagnostic approaches [10]. The risk is increased in extremely-low-birthweight (ELBW) infants in populations with a high prevalence of CMV-IgG seropositivity [11]. In the United States and in Western Europe, about 40–60% of women of reproductive age are CMV-seropositive, but this percentage is higher in Asia and Africa (up to 90%) [12].

Most of these CMV-seropositive mothers (up to 96%) undergo reactivation specifically in the mammary gland during lactation and shed the virus in BM for several weeks after delivery without showing any signs of infection [11]. Little or no virus is detected in colostrum. However, CMV DNA is increasingly detected in BM, with levels peaking at 2 to 8 weeks of lactation and declining in the subsequent weeks, often to minimal levels [5].

**Figure 2:**
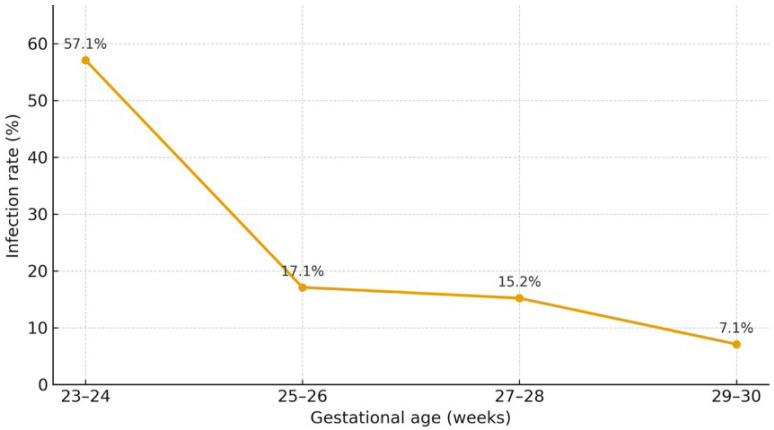
Rate of acquisition of CMV infection through the mother’s breastmilk by gestational age [14].

A recent meta-analysis estimated that CMV infection occurs in 16.5% of preterm infants born to CMV-seropositive mothers (10–26%), while CMV shedding in BM is detected in the majority of seropositive women (71–87%) [13]. Breastmilk–acquired infection affects approximately one fifth of exposed infants and is associated with a 1.6-fold higher risk of CMV acquisition compared with formula feeding [1], with gestational age acting as an important modifying factor, as shown in Figure 2. This could be related to the immaturity of premature infants’ immune systems and a lack of protective maternal antibodies, as these generally are transferred to the fetus only in the third trimester of pregnancy [2].

In addition to breastmilk, CMV-seropositive women may shed virus in genital secretions, allowing early postnatal acquisition during intrapartum exposure (within the first 4–6 weeks of life), particularly when rupture of membranes is prolonged [1,7].

Although CMV can be transmitted via blood products [9] because of its latency in bone marrow progenitor cells and monocytes [7,15], transfusion-associated postnatal infection is currently rare as a result of widespread preventive measures [7,16].

## 3. Case Series of Postnatally Acquired CMV

We describe a series of five cases of postnatally acquired CMV infection (pCMV) in newborns admitted to our unit for different reasons over the last 4 years, with these cases accounting for 0.5% of total admissions and 2.2% of admissions of preterm VLBW newborns (<32 weeks of gestation and under 1.500 g in weight). The infants’ clinical characteristics are shown in Table 1. All infants were born prematurely, with an average gestational age at delivery of 28.2 weeks. In all cases, labour began spontaneously. The details of the cases are as follows.

Our institution manages over 3000 deliveries per year and strongly supports breastfeeding. In the absence of maternal milk, formula feeding is adopted because no donor human milk bank is available, but the use of fresh maternal breastmilk is routinely encouraged. All blood-product transfusions are CMV-negative and leucocyte-depleted, following the standard of care. Currently, we are implementing the recommendation for screening of preterm infants, as suggested by the European Congenital Cytomegalovirus Initiative (ECCI) [17], but such screening was not yet a standard of care in the evaluated period. There is no specific protocol for timing of CMV testing in suspected postnatal infections, but we usually evaluated CMV DNA in urine and blood simultaneously in critical patients (those with severe and persistent thrombocytopenia, leukopenia, and/or neutropenia). Otherwise, we usually evaluated it in urine and then in blood in the case of a positive result.

### 3.1. Case 1

V.I., a late preterm female, was born at 35 + 1/7 weeks of gestation. CMV serology was not evaluated in the mother. Due to a prenatal diagnosis of duodenal atresia, she underwent abdominal surgery on the second day of life (DOL) without complications. During routinary examinations, bilateral parenchymal abnormalities, specifically hyperechogenic periventricular matter and subependymal cysts, were highlighted on cerebral ultrasound. As a result, further examinations were conducted to exclude congenital infection. The urine was positive for CMV DNA was positive on DOL51, so the blood was also evaluated on DOL53 and found to be positive. Further investigations showed normal transient evoked otoacoustic emissions (TEOAE) and automatic brain response (ABR). Dried spots collected on DOL3 were negative for CMV DNA. This case was diagnosed as a postnatally acquired infection in a healthy baby who did not need any treatment. Other intrauterine infections were excluded, and the etiology of the abnormal cUS findings was not clarified. We followed up with this child until she reached 12 months of age and found normal neurodevelopment and no other sequelae.

### 3.2. Case 2

P. C. born preterm at 28 weeks of gestation from a dichorionic diamniotic twin pregnancy. The mother was susceptible to CMV throughout the pregnancy. He was admitted to the NICU, where he needed mechanical ventilation for 7 h, then non-invasive ventilation for 7 days. He developed *Escherichia coli*-associated sepsis secondary to a central line-associated bloodstream infection (CLABSI), which was treated with oxacillin and amikacin for 14 days, with normalization of inflammatory markers and clinical restoration. Because of a persistent neutropenia with neutrophil count between 500/μL and 900/μL in several evaluations without any other alterations of hematological parameters or other blood tests, he was tested for several infections, including CMV. On DOL 30, CMV DNA was detected in the urine and blood, which was also IgM-positive. The neutropenia progressively spontaneously resolved, reaching normal values within a week after the diagnosis of CMV infection. Audiological, ophthalmological, and neurological examinations were completely normal. A postnatally acquired infection was diagnosed and did not require any medications. We followed up with this child until he reached 18 months of age, and we found normal psychomotor development and no other sequelae.

### 3.3. Case 3

G.C. was born preterm at 27 + 5 weeks of gestation, at VLBW. Her mother was seropositive for CMV in the first and early third trimesters. The baby needed mechanical ventilation for 4 days and non-invasive ventilation for another 25 days. G.C. exhibited a feeding intolerance beginning at DOL15, with vomiting and abdominal distension. In addition, beginning at DOL20, G.C. exhibited a severe thrombocytopenia (platelet count less than 20,000/μL since DOL24), requiring multiple platelet transfusions without improvement. The urine and blood were positive for CMV DNA on DOL27. CMV DNA was also detected in maternal milk evaluated on DOL 30, confirming the diagnosis of pCMV. The baby was treated with oral valganciclovir for 28 days (until CMV DNA was undetectable in the blood). Dried blood spots collected on DOL3 were negative for CMV DNA. Seven days after this diagnosis, with persistent leukopenia (white blood cells below 2000/μL and 3000/μL in several evaluations), she developed life-threatening sepsis associated with Enterobacter. Delayed psychomotor development was diagnosed at age 6 months (Griffiths DS 85, developmental age 3 months); no other sequelae were found.

### 3.4. Case 4

E.M. was born at 25 + 6 weeks of gestation, with a weight of 550 g at birth. He underwent prolonged mechanical ventilation until almost DOL100 and consequently developed severe bronchopulmonary dysplasia (BPD) with secondary pulmonary hypertension, which required medical therapy (he was dismissed at home with heated high-flow ventilation). In addition, E.M. had several episodes of bacterial sepsis during his stay. At DOL60, he showed a clinical worsening, followed by severe cholestatic hepatitis (maximum direct bilirubin 7.3 mg/dL), leukopenia (white blood cells below 2140/μL), thrombocytopenia (platelet value below 11,000/μL). CMV-specific IgM was positive, and CMV DNA was detected in both the urine and the blood (in the first week of life, the urine and DBS both tested negative for CMV DNA). Because of his severe clinical conditions, E.M. was given oral treatment with valganciclovir for 56 days, until biological samples (blood and urine) tested negative for CMV DNA. Nevertheless, the hematological findings normalized after 5 days of treatment and the hepatic function improved after two weeks and slowly normalized after 6 weeks. We considered discontinuing the antiviral therapy when regression of symptoms was demonstrated but decided to continue for another 2 weeks, as CMV DNA was still detectable. He presented with retinopathy of prematurity (ROP) stage III, which was treated with laser therapy. He was discharged home at 2 months corrected age without any ventilatory support. Delayed psychomotor development at 6 months (Griffiths DS 99, developmental age 4 months) was diagnosed, along with associated visual impairment, but the audiological evaluation was normal.

### 3.5. Case 5

O. M. was a preterm baby born at 26 + 3/7 weeks of gestation, with ELBW. She was treated with non-invasive ventilation until DOL65. She also had two different episodes of late-onset sepsis (associated with *Staphilococcus epidermidis* and *Streptococcus agalactiae*), which were treated with specific antimicrobial therapy. On DOL24, in the absence of maternal screening for CMV serology, CMV-specific IgM was positive and CMV DNA was detected in the urine and blood, although no symptoms were present. We also tested dried blood spots collected in the third DOL for CMV DNA, with a negative result. Because of this asymptomatic presentation, O.M. did not require any therapy. We diagnosed an acquired asymptomatic infection. During follow-up, delayed psychomotor development was identified at age 6 months (Griffiths DS 100) and was confirmed on subsequent evaluations; follow-up also revealed suspected autism spectrum disorder at 17 months of age.

In summary, our case series shows how pCMV manifestations are heterogenous, ranging from completely asymptomatic conditions to life-threatening disease. In fact, in three patients (60%), the infection was asymptomatic, while in the other two (40%), who had lower birthweights, pCMV can be considered a triggering condition that led to severe systemic infections. In one patient, feeding intolerance leading to NEC was the first subtle sign of pCMV. Moreover, 3 of 5 patients presented with hematological disorders (thrombocytopenia and/or leukopenia), which in two patients led to sepsis and in one was associated with hepatitis. Therefore, we experienced a wide spectrum of clinical presentation and encountered difficulties in the management of more complex patients because of a lack of clear recommendations.

## 4. Clinical Features in the Acute Phase and Short-Term Outcomes

The great majority of postnatally CMV-infected full-term, healthy newborns usually present with an asymptomatic or very mild clinical course, possibly due to the protection conferred by maternal IgG that passed through the placenta [2]. The occurrence of clinical manifestations after pCMV varies from 0% to 75%, with higher percentages in infected newborn of lower GA [12]. Nonetheless, the incidence of clinically apparent infection is estimated to be 14–50% among very premature (gestational age < 32 weeks) and/or very low birthweight (VLBW, <1500 g) infants fed BM from CMV-seropositive mothers [4], with disease typically manifesting in the second or third month of life [18]. The rate of detection of pCMV in neonatal intensive care units (NICUs) has increased in recent years due to the improving survival of extremely preterm infants and increasing awareness among clinicians [19].

Preterm infants are particularly vulnerable due to their immature immune systems and a lack of protective maternal antibodies, as these generally are transferred to the fetus only in the third trimester of pregnancy [2]. Levels of passively acquired antibody decline rapidly in this category of newborns [20], and this may result in a wide spectrum of clinical manifestations ranging from mild symptoms to life-threatening multiorgan failure.

**Figure 3:**
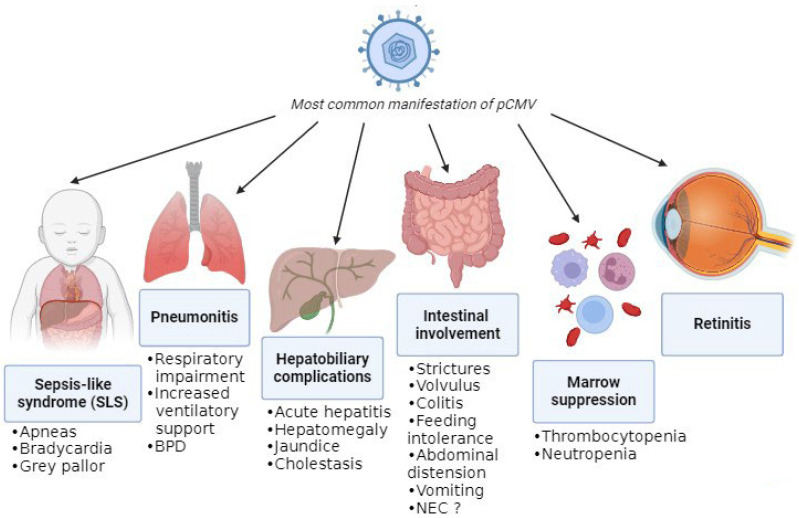
Most common manifestations of pCMV.

The absence of pathognomonic clinical features complicates attribution of disease to pCMV, as its manifestations closely resemble those of other infections and prematurity-related conditions. The most common manifestations are summarized in Figure 3.

A recent review of pCMV in preterm infants highlighted that gastrointestinal involvement was the most frequent clinical presentation, with symptoms including feeding intolerance, abdominal distension, hepatomegaly, and vomiting [3]. One proposed mechanism for the gastrointestinal complications of pCMV infection is increased intestinal permeability, which may facilitate bacterial translocation and amplify the inflammatory response [21]. Subsequently, this can lead to manifestations like strictures, volvulus, and colitis (presenting with bloody stool and an abnormal abdominal exam or radiographs) [22,23,24]. Moreover, viral persistence in the intestinal mucosa could lead to NEC, although it is difficult to confirm pCMV-mediated disease. Monitoring CMV viral load in infants with NEC and histological analysis of resected bowel specimens may help to clarify the role of pCMV. Despite reports of a twofold increased NEC risk with higher viral exposure [21], cumulative evidence indicates that pCMV is unlikely to be a significant etiological factor [4]. Additionally, infants with pCMV can experience hepatobiliary complications such as hepatosplenomegaly, acute hepatitis with impaired liver function and increase in liver enzymes, jaundice, and cholestasis [25,26].

Another common manifestation is hematological disorders due to bone-marrow suppression, such as thrombocytopenia (i.e., platelets < 150,000 cells/μL) and neutropenia (i.e., absolute neutrophil count < 1000 cells/μL), which are frequently observed either in isolation [26] or in association with other clinical features [27,28]. Notably, significant thrombocytopenia (<100,000 cells/μL), which was in some cases refractory, was present in approximately 50% of infected infants [29]; for that reason, if significant thrombocytopenia is detected, pCMV infection must be suspected. Moreover, there was a single case of secondary hemophagocytic lymphohistiocytosis triggered by pCMV infection, although this occurred in an infant born at 35 + 5 weeks of gestation and with a birthweight of 1840 g [30].

A relatively frequent presentation in preterm infants is the so-called sepsis-like syndrome (SLS), which closely resembles bacterial sepsis [25]. SLS occurs in approximately 1.4% of infected very-low-birthweight infants [27]. It is defined by the presence of three or more of the following clinical features in the absence of a positive blood culture: bradycardia or tachycardia; apnea or increased oxygen or the need for ventilatory support (including invasive or noninvasive ventilation, nasal CPAP, or high-flow nasal cannula); hypotonia or seizures; irritability or lethargy; and poor skin perfusion or prolonged capillary refill time (>3 s) [31]. A mild increase in C-reactive proteins (CRP) can be detected through blood examination [5,6,8,20,25,32,33,34,35,36,37].

Another frequent clinical feature of pCMV is pneumonia, which can lead to respiratory impairment [14,25]. In fact, CMV can affect the lungs directly through infection, with manifestations including necrotizing pneumonia and fibrosis, but it can also have indirect effects due to the inflammatory response. These combined effects can result in deterioration in respiratory status and subsequently in an increased need for ventilatory support or supplemental oxygen (≥10% above baseline), leading to prolonged mechanical ventilation and a longer hospital stay [27]. Respiratory impairment and thrombocytopenia were the findings most associated with infection that resulted in more exposure to mechanical ventilation and longer duration of hospitalization [29].

pCMV infection in extremely preterm infants has not been associated with meningitis or encephalitis. Nevertheless, in the future, CMV DNA may be detected in cerebrospinal fluid more frequently due to the use of multiplex PCR panels that include CMV. The clinical significance of CMV detection in CSF should therefore be interpreted in the context of the infant’s overall clinical presentation [38]. Furthermore, retinitis is rare in infants with postnatally acquired CMV infection. Nevertheless, retinal screening is recommended in viremic infants, as the presence of CMV-related retinitis constitutes an indication for antiviral treatment. Retinal hemorrhages may reflect underlying thrombocytopenia rather than active CMV retinitis, underscoring the need for expert ophthalmological assessment [38].

Generally, these symptoms are mostly self-limiting, but in some cases, they require symptomatic therapy and/or antiviral treatment. They rarely lead to death [7,20,24,39,40,41], as occurred in a recent case report of a former 25-week-old premature infant, fed with her mother’s milk, who was diagnosed with CMV pneumonitis only postmortem and as a result had not received antiviral treatment [42]. Furthermore, a recent cohort study of Taiwanese infants (both premature and term) with pCMV described 2.8% of mortality as CMV-associated after all other potential diagnoses had been carefully considered and ruled out [43].

Table 2 summarizes the main features of reported cases in cohort studies published since 2000.

As suggested by Bimboese P et al. [55], a categorization of infected infants based on clinical features could be useful. They defined three groups, considering five symptom categories (infection, bone-marrow suppression, gastrointestinal or respiratory symptoms, and general appearance): asymptomatic, mild (symptoms in one or two of the five categories ± abdominal distension without any major clinical deterioration), and severe (sepsis-like symptoms or symptoms in at least three of the five categories). This categorization could be useful for the management of infected patients in clinical settings.

## 5. Diagnosis

The gold standard for the diagnosis of pCMV should be a CMV-positive sample (blood, urine, CSF, nasopharyngeal aspirate or bronchoalveolar lavage, and/or saliva tested for CMV DNA by PCR) after a negative sample collected in the first 21 days of life [38]. Without this test, it could be challenging to differentiate between a congenital (cCMV) and a postnatal (pCMV) infection if the virus is detected after the first 3 weeks of life, as that is the commonly accepted cutoff time for the diagnosis of a congenitally acquired infection [17]. Retrospective diagnosis of cCMV requires the identification of the virus in dried blood spots, a method with pooled sensitivity and specificity of 84.4% and 99.9%, respectively [61].

Maternal serological screening during pregnancy is spreading as a standard of care in many countries, such as Italy [62], while screening of infants and BM is usually not performed. CMV testing is done at the clinician’s discretion, prompted by specific signs or symptoms in the infant. Currently, CMV testing is mainly performed when there is no clinical improvement after first-line antibiotic treatment, evidence of persistent marrow suppression, and/or persistent cholestasis. Notably, CMV testing should be included in “late-onset sepsis evaluation” and paired with bacterial blood cultures in cases of nonspecific instability in VLBW, breastmilk-fed infants beyond DOL 21, as recognition of CMV infection may spare infants unnecessary prolonged antibiotic treatment [29].

Preterm infants are currently not routinely screened for CMV infection, even though a recent European consensus recommends testing for CMV DNA in every preterm infant born before than 32 weeks’ gestation or at very low birthweight (<1500 g) in order to differentiate between congenital and a postnatal infections, with the latter recognized via a positive test for CMV DNA (especially after 21 DOL) with a previous negative sample [17]. Nevertheless, there is no consensus on whether to collect weekly saliva samples after a negative CMV DNA PCR at birth (which excludes a cCMV infection) to monitor at-risk infants in NICU settings, such as those born at less than 32 weeks gestational age or VLBW infants. Routine monitoring of CMV DNA levels in VLBW preterm infants with breastmilk–acquired infection may allow earlier identification of pCMV and help identify infants at risk of severe disease, potentially preventing or attenuating the development of serious clinical manifestations [38,63]. Screening using salivary samples to detect CMV DNA has been shown to be highly sensitive [37,38,64,65], except in one study, where the sensitivity was 30% (6.7–65.2%), a result that may be related to inadequate saliva collection from VLBW infants [57]. Ideally, saliva swabs require no skills to obtain and can be collected by placing a swab between the cheek and jaw, then rotating it for 5 s on both sides, but VLBW infants can present with only small amounts of saliva, making collection more difficult [66]. Swabs can be placed in viral transport media (1–3 mL) or air-dried before transport. Collecting saliva is a painless and quite accurate method of detecting the virus, although the sample must be collected before breastfeeding or at least 1 h after to avoid false-positive results [65]. Thus, any positive result should be confirmed via a urine sample [17]. Moreover, saliva collection is acceptable to parents [37,38,64,65].

However, this approach—commonly referred to as “pre-emptive screening” when it is used in transplant recipients—has not yet been evaluated for clinical benefit or cost-effectiveness in the context of postnatally acquired CMV infection. Further studies addressing the natural history and long-term outcomes of pCMV are required to support its implementation, although batch-testing strategies may help to reduce costs [38,67].

CMV viral load in urine is lower in infants with pCMV (1.0 × 10^5^ copies/mL) compared with infants with cCMV (8.5 × 10^6^ copies/mL) [68]. Furthermore, there are no robust data describing any association between CMV viral load and adverse clinical outcomes in pCMV [38]. CMV DNA is also frequently detected by PCR in respiratory secretions (nasopharyngeal aspirate or bronchoalveolar lavage), blood, and cerebrospinal fluid.

## 6. Long-Term Outcomes

The great majority of term newborns acquiring CMV infection postnatally have no long-term consequence, although the potential role of pCMV infection in causing adverse long-term outcomes in preterm infants is not well defined and remains a subject of debate [4].

A recent systematic review indicated that preterm infants with postnatally acquired CMV infection, particularly those with symptomatic disease, may be at increased risk of long-term pulmonary and neurodevelopmental sequelae compared with pCMV-negative infants, although these findings are inconsistent and may be influenced by gestational age at birth, variability in NICU practices, and the absence of standardized treatment guideline [4]. Nevertheless, a more recent study not included in this review found no significative incidence of neurodevelopmental impairment (NDI) in symptomatic preterm infants on follow-up until 3 years of age, although the risk of ND was higher and all developmental quotients were lower in this population than in the control group [54].

Overall, the evidence suggests a positive association between postnatally acquired CMV infection and bronchopulmonary dysplasia diagnosed at an adjusted age of 36 weeks. However, confirmation of this relationship requires large, methodologically robust prospective studies, as well as high-quality randomized controlled trials, to assess whether prevention or treatment of viral infections can modify the risk of BPD [69]. Several mechanisms have been proposed to explain this association. Similar to late-onset bacterial infections, symptomatic pCMV infection may act as an independent contributor to respiratory deterioration and BPD development through both virus-induced inflammation and direct viral lung injury, leading to prolonged mechanical ventilation and increased oxygen requirements—established risk factors for BPD [27,70].

Case reports following the long-term NDI of infants with pCMV found differing results [4]. Studies suggest that infants with postnatally acquired CMV infection are at increased risk of NDI, including global developmental delay, hypotonia, and deficits in psychomotor, language, and visuospatial domains, with some effects becoming apparent only at school age. Importantly, studies reporting no differences in neurodevelopmental outcomes generally had shorter follow-up periods, underscoring the importance of long-term surveillance. Interpretation of these findings is further complicated by multiple confounding factors, including degree of prematurity, associated comorbidities, nutritional status, socioeconomic conditions, and access to early-intervention services. Moreover, preterm infants miss critical periods of in utero brain maturation, resulting in heightened vulnerability of oligodendrocytes and of key neurodevelopmental processes, including synaptogenesis, glial proliferation, and differentiation, which may increase susceptibility to CMV-related injury [71,72].

pCMV is not associated with damage involving the central nervous system (CNS) or the sequelae that have been described after congenital infection [33]. However, an increased prevalence of lenticulostriate vasculopathy (LSV) has been described in pCMV-infected infants, perhaps as a result of necrotizing inflammation secondary to CMV infection [73]. No disadvantageous alterations in microstructural brain maturation were observed at term-equivalent age in a recent study [74]. Additionally, abnormalities seen on neuroimaging do not always translate to NDI.

Although congenital CMV infection is the leading non-genetic cause of hearing loss in infancy, most available studies indicate that postnatally acquired CMV infection does not increase the risk of permanent hearing loss in preterm infants [4]. A retrospective cohort study reported a higher rate of hearing-screening failure at hospital discharge among preterm infants with pCMV infection, a result possibly related to direct viral cytopathic effects and associated inflammatory responses similar to those implicated in congenital CMV [52]. However, failure of the initial hearing screen does not necessarily indicate permanent hearing impairment, and normal hearing may be documented with longer follow-up. While congenital CMV is a recognized cause of chorioretinitis [75], current evidence suggests that postnatally acquired CMV infection in preterm infants is not associated with an increased prevalence of ocular complications or retinopathy of prematurity (ROP) [4]. Nonetheless, isolated cases of ROP worsening during pCMV infection have been re-ported, with improvement following antiviral therapy [76]. More recently, a case of diffuse occlusive retinal vasculitis incidentally identified during routine ROP screening has also been described [77].

Symptomatic pCMV-infected infants may have lower weight gain while hospitalized, but pCMV does not have a long-term impact on weight-gain trajectory. Moreover, neither head circumference nor length seem to differ in preterm pCMV-infected infants compared with their uninfected counterparts [4].

The recognition of the most common features of pCMV infection is important to allowing clinical practitioners to promptly recognize these symptoms and identify infants for whom antiviral therapy and/or more intensive auditory and neurodevelopmental follow-up care may be indicated.

## 7. Treatment

Intravenous ganciclovir (GCV) and its oral prodrug valganciclovir (V-GCV) are recommended treatments for infants with congenital CMV infection who present with moderate-to-severe disease or central nervous system involvement, based on established guidelines [17]. In this population, antiviral therapy has demonstrated modest benefits in preventing hearing deterioration and improving neurodevelopmental outcomes at two years of age [78,79]. By analogy, similar antiviral strategies may be considered in very-low-birthweight infants with breastmilk–acquired CMV infection who develop severe CMV-related clinical manifestations. However, evidence supporting antiviral treatment in pCMV is limited to case reports and small retrospective studies, with no randomized controlled trials available and no specific guidelines currently established [8,50,63,80]. Consequently, there are no evidence-based recommendations regarding which infants with pCMV should be treated or the optimal timing of therapy initiation. Clinical decision-making is further complicated by uncertainties surrounding the true burden of infection-related morbidity and the extent to which antiviral treatment improves outcomes [59]. In addition, the safety profiles of ganciclovir and valganciclovir in VLBW infants remain poorly defined, as pharmacokinetic and safety data are scarce in this population, despite their higher risk of severe disease; both agents are known to be associated with potential toxicities in infants [78,79]. Given these limitations, the evidence base guiding pCMV management remains sparse and antiviral treatment is generally reserved for infants with severe, symptomatic disease [81].

Before antiviral treatment is initiated in infants less than 32 weeks’ gestation and weighting less than 1200 g, there should be a documented discussion with parents in-forming them that no studies are available on infants treated with this medication at lower gestational age and birthweight [78] and that it may have short-term and long-term side effects, including severe neutropenia and the risk of carcinogenicity and germline damage (to date, demonstrated only in animal studies) [38]. Thus, the decision to start antiviral treatment should be based on the severity of the clinical condition caused by CMV infection, the side effects of the treatment, any underlying conditions that may predispose to or exacerbate infection, and the viral load, as suggested recently [38].

An active CMV-related retinitis in a viremic baby is an indication for treatment, as a pCMV infection in a preterm infant coexisting with ROP can result in worsening vitro-retinal lesions [82] and prompt treatment is crucial to avoid visual impairment [77].

The infants most likely to benefit from antiviral therapy are those with confirmed severe postnatally acquired CMV disease; in these patients, treatment is aimed at suppressing active viremia and preventing progressive end-organ damage rather than modifying the course of chronic infection. Some authors have suggested monitoring CMV DNA levels in very-low-birthweight preterm infants during the breastfeeding period to enable a “pre-emptive” treatment approach when viral load increases, an approach similar to strategies used in immunocompromised patients to prevent severe CMV disease [65]. However, this approach is not currently widely accepted.

pCMV can be treated with oral V-GCV in enterally fed infants or with intravenous GCV in those who do not tolerate feeding. The dosages are the same as those currently used for cCMV for infants > 32 weeks GA: 6 mg/kg twice daily intravenously of GCV or 16 mg/kg twice a day orally of V-GCV [78,79].

Careful monitoring for adverse events is mandatory during antiviral therapy. Neutropenia has been reported in up to 63% of infants treated with ganciclovir and 19% of those receiving valganciclovir [78,79]; therefore, complete blood counts should be obtained at least weekly throughout treatment. If the absolute neutrophil count falls below 500/μL, antiviral therapy may be temporarily discontinued until levels recover to above 750/μL or continued with the addition of granulocyte colony-stimulating factor in severely symptomatic infants. Liver function and renal function should also be monitored weekly, and dose adjustment is required in the presence of renal impairment, as both ganciclovir and valganciclovir are primarily renally excreted. Reports of hepatotoxicity related to antiviral treatment in postnatally infected preterm infants are rare; only one case of elevated transaminases and cholestasis has been described [8], while other series have reported no adverse events attributable to ganciclovir or valganciclovir, with stable hematological and hepatic parameters during treatment. Monitoring viral load weekly during treatment via testing for CMV DNA in blood could be useful as a way to assess antiviral efficacy. Full viral suppression usually leads to disease resolution, even if timing depends on the initial level of viremia and the severity of end-organ disease.

Careful consideration should therefore be given to treatment duration. Antiviral therapy for a minimum of two weeks has been proposed, with weekly monitoring of CMV viral load. If clinical symptoms persist and viral suppression is incomplete, treatment may be extended in additional two-week intervals. Because antiviral resistance has been reported after prolonged exposure, treatment durations exceeding eight weeks are not recommended [38]. In cases of symptomatic disease that fails to respond to antiviral therapy, the presence of an underlying immunodeficiency, such as HIV infection or severe combined immunodeficiency, should be investigated.

No studies have evaluated the efficacy of alternative antiviral agents for the treatment of symptomatic postnatally acquired CMV infection. Given the protective role of CMV-specific IgG, one study explored the use of CMV-specific hyperimmune globulin in extremely-low-birthweight infants with established CMV disease, particularly those born at ≤24 weeks’ gestation [83]. Further research is required to confirm these findings and to determine whether this approach has a role in routine clinical practice. The use of corticosteroids has been reported in few cases, usually in association with antiviral therapy and mainly to treat persistent thrombocytopenia [3].

**Figure 4:**
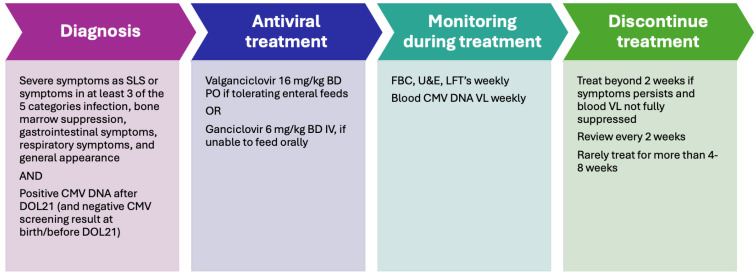
The management of pCMV [38,55]. FBC: full blood count; U&E: urea and electrolytes; LFT: liver function test; VL: viral load.

The management of pCMV is summarized in Figure 4.

## 8. Breastfeeding and CMV

### 8.1. Techniques for Reducing the Risk of CMV Transmission via Breastmilk

**Figure 5:**
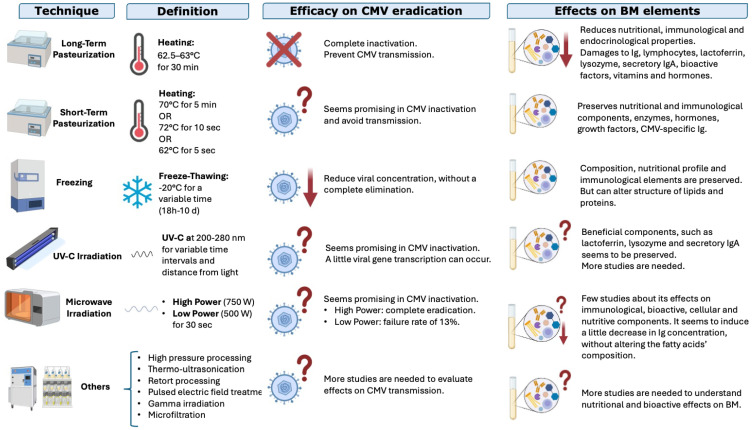
Techniques used to treat breastmilk, their efficacies in reducing the risk of CMV transmission, and their effects on nutritional and functional elements in BM; ↓ indicates a reduction, ? indicates that the result is not well known; X indicates inactivation (modified from Bardanzellu F. et al. [12]).

Several techniques can be used to treat BM and to reduce the risk of CMV transmission through this valuable superfood: some of them are able to completely eliminate the risk of CMV transmission but on the other hand can damage functional or nutritional substances. The options are analyzed below with the pros and cons of each; the analysis is summarized in Figure 5.

**Pasteurization:** Heat is one of the oldest and most studied methods to treat BM. We can distinguish two types of pasteurization:

a. Long-term pasteurization (“Holder” pasteurization) involves heating BM to 62.5–63 °C for 30 min and is the preferred method used by human milk banks to inactivate CMV and make BM microbiologically safe. It effectively inactivates CMV but can reduce some bioactive components of BM [37,84], such as proteins, enzymes, and vitamins (for example vitamin C, B6, or folate) [84,85,86,87,88,89,90];
b. Short-term pasteurization uses a higher temperature for a shorter time, but there is not a univocal definition: some authors use 70 °C for 5 min, others 72 °C for 10–15 s, others still 62° C for 5 s [12]. The procedure was conceived to ensure microbiological safety while preserving some of the bioactive factors. Recent studies found that the duration of the process had a greater impact on the qualitative composition of BM than the temperature used [91].

**Freezing:** Freezing at −20 °C for a variable time (from 18 h to 10 days or more, depending on authors) can significantly reduce the viral load [37], even if the virus is not completely eliminated [12]. Some authors highlighted a relationship between freezing duration and viral load. However, this method can alter the structures of lipids and proteins [37,92,93,94].

**UV-C Irradiation:** UV-C irradiation (200–280 nm for several time intervals) is a promising method for inactivating CMV in BM while preserving its nutritional components and even lactoferrin, lysozyme, and secretory IgA [37]. However, further studies are needed [10,37], especially to clarify the appropriate dose and conditions for an optimal irradiation [95,96].

**Microwave Irradiation:** Irradiation at 750 W for 30 s has been recently studied [12]. It can inactivate CMV in BM, but the effects on its functional properties and eventual side effects of undesired heat are not yet known [97].

More recent but less studied technologies include high-pressure processing, thermo-ultrasonication, retort processing, pulsed electric-field treatment, gamma irradiation, and microfiltration. More studies are needed to understand their effects on CMV viral load and on nutritional, bioactive, and functional substances in BM [98].

### 8.2. Benefits of Breastmilk in the NICU

BM is the optimal nutrition for newborns, especially preterm infants, because it provides not only essential nutrients but also unique immune, anti-inflammatory, and developmental benefits [12,84,99]. Its complex and variable composition includes bioactive substances such as growth factors, immunoglobulins, enzymes, immune cells, and human milk oligosaccharides, which change over time and adapt to infants’ needs [84,99]. Milk from mothers of preterm infants is especially tailored [99], containing higher protein and mineral levels and more easily digestible fats [84]. Breastfeeding improves short-, medium-, and long-term health outcomes, reducing risks of conditions such as necrotizing enterocolitis, sepsis, retinopathy of prematurity, infections, chronic diseases, obesity, and sudden infant death syndrome, while supporting growth and neurodevelopment [99]. It is also cost-effective and emotionally beneficial [100]. Consequently, ESPGHAN recommends maternal BM as the first-choice nutrition for preterm infants, with fortified donor human milk as the alternative when maternal milk is unavailable [99].

### 8.3. Current Management of BM for Prevention of pCMV

**Figure 6:**
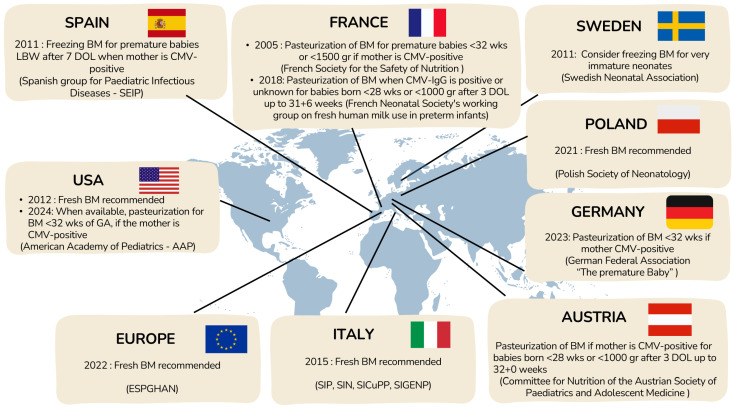
Current management for prevention of pCMV transmission through BM.

Currently, there is no standardized algorithm for deciding to treat human milk from CMV-IgG seropositive mothers of premature neonates. Each NICU follows its own guidelines based on national recommendations, when those exist, balancing the benefits of feeding extremely preterm newborns with their mothers’ milk and the risks of potential viral transmission. Available national recommendations are summarized in Figure 6.

In 2012, the AAP concluded that the benefits of fresh maternal BM outweighed the risk of CMV transmission [100], but in 2024, it recommended CMV screening for mothers of infants born before 32 weeks’ gestation and treatment of milk from CMV-IgG–positive mothers, while still not supporting withholding maternal milk due to insufficient evidence [101]. In contrast, ESPGHAN 2022 guidelines advise against routine pasteurization of maternal milk because it reduces bioactive components, despite their acknowledgment of potential CMV risks [99].

Across Europe, practices vary widely due to heterogeneous national recommendations [102]. Some countries (e.g., Spain [103], Sweden [104]) favor freezing milk to reduce viral load, while others (Austria [105], Germany [106,107], France [108]) recommend pasteurization for extremely preterm or very-low-birthweight infants. Poland [109] and Italy [110] emphasize the benefits of fresh BM and discourage routine pasteurization. Overall, there is no consensus, reflecting a balance between minimizing the risk of CMV transmission and preserving the well-established benefits of unprocessed maternal BM for preterm infants.

In some cases, when newborns cannot be fed with their mothers’ milk due to insufficient lactation or conditions contraindicating breastfeeding with their own mothers’ milk, human donated milk is an alternative. Human milk banks collect and process samples from volunteer donors, who are accurately selected and screened, to provide BM for these vulnerable newborns. To avoid viral or bacterial transmission, including CMV, donor human milk must undergo long-term pasteurization. Alternative treatments are not recommended, as indicated in the Consensus published in 2019 by the European Milk Bank Association (EMBA) [111].

## 9. Other Strategies for the Prevention of CMV Transmission in Preterm Babies

Efforts have been made to decrease the risk of CMV transmission via blood-product transfusion in high-risk patients such as newborns, with examples including using blood products from donors that are CMV seronegative and filtering out white blood cells that carry CMV using leukoreduction or both [7,112]. Exploring alternative methods to make milk non-infectious is also worthwhile; such methods include the use of anti-CMV immunoglobulins added to the milk or leukofiltration of the milk, as is currently done for blood [10,37].

The administration of CMV-specific hyperimmune globulin to ELBWIs could prevent pCMV infection via blood transfusion, especially in ELBWIs of ≤24 weeks’ gestation [83]. However, this potential protective effect was evaluated only in a single, older study (although a randomized, placebo-controlled, double-blind trial), possibly because the current treatments used for blood products are effective, in terms of both safety and cost.

Furthermore, the neutralizing capacity of monoclonal antibodies in preventing viral transmission was assessed for preventing CMV infection in a phase 2a trial with CMV-seronegative recipients of kidney transplants from CMV-seropositive donors [113]. Promising results in this population have not led yet to similar trials to reduce maternal–fetal transmission.

In terms of prevention, lactoferrin—a natural component of BM—has shown promise. It exhibits neutralizing activity against CMV by blocking viral entry into cells, as demonstrated experimentally both in vitro and in vivo [114]. However, a recent study revealed no significant difference in lactoferrin concentrations between transmitter and non-transmitter seropositive CMV mothers, and its levels in maternal milk were found to be insufficient for effective prevention [115]. In conclusion, further research is needed to determine whether higher concentrations of lactoferrin could prevent CMV transmission via breastfeeding.

The development of a vaccine is also being considered as a strategy to prevent postnatal transmission of CMV infection. A study in Uganda examined CMV-specific immunoglobulin G responses in mothers at delivery and their infants’ CMV status at six months of age. The results suggested that elevated glycoprotein B-specific IgG levels might provide partial protection against pCMV infection. These findings highlight the importance of further research into glycoprotein B antigens as a potential basis for a CMV vaccine [116].

Maternal screening is another way to prevent eventual negative outcomes of pCMV. In fact, screening mothers for CMV antibodies can identify seropositive women at risk of non-primary infection and of subsequently shedding the virus in BM and alert clinicians on eventual signs or symptoms of pCMV infection in their very preterm babies [37].

## 10. Future Directions

The overall clinical burden of postnatally acquired CMV infection remains poorly defined, largely because of the limited availability of long-term outcome data. At present, no major scientific society has issued formal guidelines or consensus recommendations regarding the prevention or management of pCMV infection in preterm infants. Consequently, the clinical course and management of affected infants may vary considerably depending on local practices and provider experience.

The only widely endorsed recommendation is early CMV screening in very preterm and very-low-birthweight infants to distinguish congenital from postnatal infection [17]. While this approach should be broadly implemented, it is insufficient on its own to guide comprehensive pCMV management. When saliva is used for diagnostic testing, careful collection of an adequate sample volume is essential to minimize the risk of false-negative results.

A pre-emptive screening strategy, defined as systematic testing to identify CMV infection at an early, often asymptomatic stage, rather than symptom-driven testing as routinely performed in transplant populations, may prove useful for epidemiological studies in preterm infants. Such an approach could help clarify the incidence and long-term consequences of pCMV infection, disentangle its effects from those of extreme prematurity, and determine whether antiviral therapy modifies outcomes. Earlier identification of pCMV may also prevent unnecessary and potentially harmful antibiotic exposure, given that pCMV frequently mimics bacterial infection. Moreover, the absence of routine CMV testing during evaluations for late-onset sepsis in VLBW infants may result in misclassification of symptomatic pCMV cases as culture-negative sepsis, thereby resulting in an underestimate of the true impact of the disease on long-term outcomes.

At present, initiating antiviral therapy before the onset of symptoms—using strategies analogous to those applied in adult solid-organ-transplant recipients—does not appear justified, although this approach may warrant future investigation to assess its potential role in reducing long-term sequelae.

Further studies are needed to better define the benefits and risks of ganciclovir and valganciclovir therapy in preterm infants, to evaluate the use of alternative antiviral agents to treat symptomatic postnatally acquired CMV infection, and to assess the role of CMV-specific hyperimmune globulin for prevention or treatment, particularly in extremely-low-birthweight infants. Finally, classifying infected infants according to clinical severity may improve prognostic stratification, while large prospective studies with long-term follow-up are essential to fully characterize the outcomes associated with pCMV infection.

## 11. Conclusions

Postnatal CMV infection may lead to symptomatic end-organ disease and/or sepsis-like syndrome in approximately 5% of extremely preterm infants, with breastmilk representing the primary source of transmission. Accordingly, pCMV infection should be considered in VLBW and ELBW infants born to CMV-seropositive mothers who present with severe clinical manifestations, particularly culture-negative sepsis, to allow timely diagnosis and appropriate management. Beyond this established association, our review underscores the need for robust evidence to guide clinical decision-making in this vulnerable population.

Improved understanding of the epidemiology of pCMV infection is essential to support evidence-based management strategies. In particular, clearer characterization of severe acute disease and long-term outcomes in preterm infants is needed. Such data would help identify which infants are most likely to benefit from antiviral therapy, an area that requires further evaluation in larger cohorts to better define indications, dosing, and treatment duration, especially in infants born before 32 weeks’ gestation. Existing registries for congenital CMV infection, such as CCMVNET (https://ccmvnet.org), could be expanded to include postnatally acquired infections, thereby facilitating higher-quality diagnosis, optimized supportive care, more consistent use of antiviral therapy when indicated, and assessment of the potential role of pre-emptive treatment strategies in high-risk neonates.

Key recommendations are summarized in Table 3.

Figures were created with Biorender.com or using iStock (https://www.istockphoto.com/it; accessed on 20 November 2025).

## Figures and Tables

**Table 1:** Clinical characteristics of postnatally infected infants.

	1	2	3	4	5
GA at birth	35 + 1/7	28 + 5/7	27 + 5/7	25 + 6/7	26 + 3/7
GA at diagnosis	42 + 3/7	39 + 2/7	31 + 4/7	33 + 6/7	30 + 0/7
Type of delivery	SD	CS	CS	SD	SD
Sex	Female	Male	Female	Male	Female
Body weight at birth (g)	2090	1230	850	550	950
Head circumference (cm)	32.5	27	22	20.5	23.5
Signs and symptoms at diagnosis	Abnormal cUS	Isolated neutropenia	Feeding intolerance, possible NEC, severe thrombocytopenia, leucopenia	Hepatitis (abnormal liver function tests), persistent thrombocytopenia, leucopenia	No significant abnormalities
Fed by maternal milk	Yes, but not exclusively	Yes, but not exclusively	Yes, exclusively	Yes, but not exclusively	Yes, but not exclusively
CMV DNA in maternal milk	N/A	N/A	Positive	N/A	N/A
CMV DNA in urine (IU/mL)	24,919	39,574	2,030,000	2,698,486	268,729
CMV DNA in blood (IU/mL)	3568	4356	187,000	764,771	37,148
CMV DNA in DBS	Negative	Negative	Negative	Negative	Negative
Treatment	No	No	Valganciclovir	Valganciclovir	No
Treatment Duration	No	No	28 days	56 days	No
Outcome	Normal	Normal	Delayed psychomotor development at 6 months (Griffiths DS 85, developmental age 3 months)	Delayed psychomotor development at 6 months (Griffiths DS 99, developmental age 4 months), BPD, ROP stage III treated with laser therapy, visual impairment	Delayed psychomotor development at 6 months (Griffiths DS 100), suspected autism spectrum disorder at 17 months

GA: gestational age; DBS: dried blood spot; SD: spontaneous delivery; CS: caesarean section; cUS: cranial ultrasound; NEC: necrotizing enterocolitis; N/A: not available; DS: developmental scale; BPD: bronchopulmonary dysplasia; ROP: retinopathy of prematurity.

**Table 2:** Main features of reported cases from cohort studies published since 2000 that include more than five preterm infants with postnatally acquired CMV infection.

	n. of Patients	Main Clinical Features at Presentation	Major Laboratory Abnormalities at Presentation	Significant Radiological Abnormalities at Presentation
Hamprecht K et al., 2001 (Germany) [44]	33	SLS (25%), myoclonia (12%)	neutropenia (88%), thrombocytopenia (12%)	not reported
Maschmann J et al., 2001 (Germany) [26]	33	SLS (48.5%)	hepatopathy, neutropenia, thrombocytopenia (48.5%)	not reported
Jim W-T et al., 2004 (Taiwan) [45]	6	SLS (% not reported)	hyperbilirubinemia (% not reported)	not reported
Vollmer B et al., 2004 (Germany) [46]	22	SLS (18%), petechia (4%)	hepatopathy (23%), thrombocytopenia, and neutropenia (555%),	not reported
Meier J et al., 2005 (Germany) [47]	13	SLS (7.7%), jaundice (7.7%)	hepatitis with conjugated hyperbilirubinemia (7.7%), neutropenia (7.7%)	not reported
Neuberger P et al., 2006 (Germany) [28]	40	not reported	thrombocytopenia, neutropenia, elevated C-reactive protein, cholestasis (7%)	not reported
Jim W-T et al., 2009 (Taiwan) [48]	8	SLS (25%), prolonged jaundice (12.5%), pneumonitis (12.5%)	thrombocytopenia and neutropenia (12.5%)	not reported
Capretti M et al., 2009 (Italy) [49]	9	SLS (33%),	neutropenia (56%), conjugated hyperbilirubinemia (56%)	not reported
Josephson CD et al., 2014 (USA) [7]	29 (5 died)	NEC (10%), SLS (3%)	elevated liver enzymes (7%), conjugated hyperbilirubinemia (7%), thrombocytopenia (3%), neutropenia (3%)	not reported
Mehler K et al., 2014 (Germany) [50]	11	SLS (55%), respiratory failure (55%)	thrombocytopenia (100%), mildly elevated CRP values (55%)	not reported
Romero-Gómez MP et al., 2015 (Spain) [5]	13 (1 died)	pneumonia (23%), hepatosplenomegaly (7.7%), jaundice (7.7%)	cholestasis (7.7%), elevated liver enzymes (7.7%)	lenticulostriate vasculopathy (31%)
Yoo HS et al., 2015 (South Korea) [11]	27 with 22 (82%) symptomatic (3 died)	19% increased respiratory support	thrombocytopenia (63%), neutropenia (44%), conjugated hyperbilirubinemia (30%), elevated liver enzymes (26%)	not reported
Kelly MS et al., 2015 (USA) [27]	328 (4 died)	SLS (18%), NEC (4%)	thrombocytopenia (66%), hyperbilirubinemia (66%), neutropenia (34%), transaminitis (16%)	not reported
Jim W-T et al., 2015 [38] (Taiwan)	14	SLS (57%)	not reported	not reported
Martins-Celini FP et al., 2016 (Brazil) [14]	24	SLS (12%)	elevated gamma GT (50%), thrombocytopenia (36%), neutropenia (18%)	not reported
Mukhopadhyay S et al., 2016 (USA) [29]	27	SLS (48%)	thrombocytopenia (52%), neutropenia (41%), cholestasis (30%), elevated liver enzymes (33%)	not reported
Gunkel J et al., 2018 (The Netherlands) [51]	74	pneumonia (3%), SLS (1%)	thrombocytopenia (1%)	LSV (36%), germinolytic cysts (15%)
Patel RM et al., 2019 (USA) [21]	33	NEC (18%)	not reported	not reported
Weimer KED et al., 2020 (USA) [52]	273	SLS (1.4%)	not reported	not reported
Garofoli F et al., 2021 (Italy) [10]	10	SLS (100%)	not reported	not reported
Hernandez-Alvarado N et al., 2021 (USA) [53]	9	SLS (55%)	thrombocytopenia (44%), renal disease (11%)	not reported
Takemoto K et al., 2021 (Japan) [54]	24	SLS (58%)	elevated C-reactive protein (82%), thrombocytopenia (74%), hyperbilirubinemia (26%), elevated liver enzymes (17%)	not reported
Bimboese P et al., 2022 (Australia) [55]	27 with 19 (70%) symptomatic (6, 22% severely symptomatic)	abdominal distension (56%) with clinical NEC (7%), respiratory deterioration (33%: apnea, new CPAP requirement, new intubation, increasing oxygen requirement); pallor (22%)	neutropenia (44%), thrombocytopenia (15%)	not reported
Minihan L et al., 2022 (Australia) [33]	48 (2 died)	abdominal distension (44%), SLS (29%), hepatosplenomegaly (13%), petechiae (13%), jaundice (10%), pneumonitis (6%)	thrombocytopenia (61%), conjugated hyperbilirubinemia (61%), elevated liver enzymes (49%), neutropenia (48%)	
Lee JE et al., 2022 (South Korea) [56]	17	not reported	elevated liver enzymes (41%), neutropenia (35%), thrombocytopenia (18%), conjugated hyperbilirubinemia (12%)	not reported
Mukhopadhyay S et al., 2022 (USA) [57]	10	respiratory impairment (20%), apnea and bradycardia (10%)	thrombocytopenia (20%), elevated C-reactive protein (10%)	None
Namba F et al., 2022 (Japan) [35]	12 (1 died)	Need for respiratory support (58%), bradycardia (42%), apnea (33%), petechia (25%)	thrombocytopenia (67%), neutropenia (33%), elevated liver enzymes (33%)	not reported
Ogawa R et al., 2022 (Japan) [58]	7 (1 died)	pneumonia (71%), SLS (14%), NEC (14%), hepatomegaly (14%)	neutropenia (86%), conjugated hyperbilirubinemia (43%), thrombocytopenia (29%), elevated liver enzymes (14%), elevated C-reactive protein (71%)	not reported
Chung ML et al., 2023 (South Korea) [34]	7 (2 symptomatic, 1 died)	respiratory impairment (29%, 2/2); SLS (14%, 1/2)	elevated liver enzymes (14%), thrombocytopenia (14%)	not reported
Chen YN et al., 2023 (Taiwan) [37]	251 (67/81 term + preterm) (140 < 90 DOL)	prolonged jaundice (32.1%); pneumonitis (0.7%), colitis (0.7%), neurological abnormalities (2.9%)	hepatitis (47.9%), thrombocytopenia (28.6%), neutropenia (0.7%)	not reported
Košiček R et al., 2023 (Slovenia) [59]	53	hepatosplenomegaly (68%), jaundice (25%)	elevated liver enzymes (53%), thrombocytopenia (51%), neutropenia (11%)	not reported
Wojciechowska D et al., 2025 (Poland) [60]	5 (1 died)	SLS with shock (20%), jaundice (40%), pneumonia (20%)	thrombocytopenia (60%), elevated C-reactive protein (40%), elevated liver enzymes (20%)	not reported

**Table 3:** Key recommendations.

**Postnatal cytomegalovirus (pCMV) infection is a relevant but often under-recognized condition in very preterm infants**, particularly those born at < 32 weeks’ gestation or with very low birthweight, and may present with sepsis-like syndrome, cytopenias, hepatitis, gastrointestinal involvement, or respiratory deterioration.
**Accurate diagnosis of pCMV requires detection of CMV DNA after the first 21 days of life following a negative early sample** in order to differentiate postnatal from congenital infection; CMV testing should be considered in culture-negative late-onset sepsis in breastfed VLBW infants.
**Breastmilk remains the optimal nutrition for preterm infants**, and current evidence does not support withholding maternal milk because of the risk of CMV transmission; however, approaches to breastmilk handling (fresh, frozen, or pasteurized) in CMV-seropositive mothers vary widely across countries due to the lack of international consensus.
**Antiviral therapy with ganciclovir or valganciclovir should be reserved for infants with severe, symptomatic pCMV disease**, while asymptomatic or mildly symptomatic infections generally do not require treatment, given the limited evidence on efficacy and the potential for drug toxicity in this population.
**Long-term outcomes of pCMV infection in preterm infants remain incompletely defined**, highlighting the need for harmonized, evidence-based guidelines on screening, breastmilk management, and treatment, as well as prospective studies with long-term follow-up.

## Data Availability

No new data were created or analyzed in this study.

## Abbreviations

The following abbreviations are used in this manuscript:

CMV	Cytomegalovirus
pCMV	Postnatal Cytomegalovirus
VLBW	Very low birthweight
ELBW	Extremely low birthweight
BM	Breastmilk
GA	Gestational age
SD	Sponteanous delivery
CT	Cesarean section
DBS	Dried blood spot
cUS	Cerebral ultrasound
NEC	Necrotizing enterocolitis
DOL	Day of life
TEOAE	Transient evoked otoacoustic emissions
ABR	Automatic brain response
BPD	Bronchopulmonary dysplasia
NICU	Neonatal intensive care unit
SLS	Sepsis-like syndrome
CSF	Cerebrospinal fluid
CRP	C-reactive protein
NDI	Neurodevelopmental impairment
CNS	Central nervous system
LSV	Lenticulostriate vasculopathy
ROP	Retinopathy of prematurity
GCV	Ganciclovir
V-GCV	Valganciclovir
FBC	Full blood count
U&E	Urea and electrolytes
LFT	Liver function test
VL	Viral load
AAP	American Academy of Pediatrics
